# Impact of nursing strategy based on the 6S management model on clinical outcomes in patients with acute myocardial infarction after Percutaneous Coronary intervention

**DOI:** 10.12669/pjms.42.7.15965

**Published:** 2026-07

**Authors:** Ying Zhang, Zhi Chen, Mengyin Gao, Zhiyin Li

**Affiliations:** 1 Ying Zhang Department of Cardiology, The Second Hospital of Hebei Medical University, Shijiazhuang, Hebei Province 050000, P.R. China; 2 Zhi Chen Medical Equipment Emergency Management Center, Hebei Provincial People’s Hospital, Shijiazhuang, Hebei Province 050051, P.R. China.; 3 Mengyin Gao Department of Emergency, The Second Hospital of Hebei Medical University, Shijiazhuang, Hebei Province 050000, P.R. China; 4 Zhiyin Li Department of Cardiology, The Second Hospital of Hebei Medical University, Shijiazhuang, Hebei Province 050000, P.R. China

**Keywords:** 6S, Acute myocardial infarction, Management model, Percutaneous coronary intervention

## Abstract

**Objective:**

To explore the impact of the 6S management model-based nursing program on clinical outcomes in patients with acute myocardial infarction (AMI) after percutaneous coronary intervention (PCI).

**Methodology:**

A retrospective analysis included clinical data from 144 AMI patients who underwent PCI at the Second Hospital of Hebei Medical University between January 2024 to October 2025. Patients were matched 1:1 to an observation group (6S nursing) and a control group (conventional nursing), with 72 cases in each group. Rehabilitation-related indicators, cardiac function indexes, activities of daily living (ADL), anxiety levels, and adverse cardiac events were compared between the two groups.

**Results:**

The observation group reported lower postoperative oral intake and ambulation times, shorter hospital stays, shorter time to resolution of ipsilateral limb swelling and longer 6-Minute Walk Test (6MWT) distances (P<0.05). Baseline Left Ventricular Ejection Fraction (LVEF), Left Ventricular End-Diastolic Diameter (LVEDD), Barthel Index (BI) and Self-rating Anxiety Scale (SAS) values showed no between-group differences (P>0.05). Post-intervention, both groups had higher LVEF and BI and lower LVEDD and SAS levels (P<0.05), with the observation group showing more favorable results (P<0.05). Compared with the control group, the incidence of adverse cardiac events such as angina pectoris and severe arrhythmia after PCI were significantly lower in the observation group (P<0.05).

**Conclusions:**

Compared with conventional nursing care, the 6S management model-based nursing program was associated with more favorable rehabilitation-related indicators, improved cardiac function, better activities of daily living, lower anxiety levels, and reduced incidences of specific adverse cardiac events in AMI patients after PCI. The 6S model optimizes inpatient outcomes by establishing standardized nursing processes, strengthening safety awareness, and ensuring the rigorous execution of rehabilitation measures.

## INTRODUCTION

Acute myocardial infarction (AMI) is one of the leading global causes of mortality and disability associated with cardiovascular diseases.^1^ The success rate of acute-phase treatment for AMI patients has improved significantly with the routine use of percutaneous coronary intervention (PCI), thereby reducing in-hospital mortality.^2^ However, as PCI alone cannot eliminate the risks of postoperative reinfarction, bleeding complications and reduced quality of life, the post-PCI period has become a critical management stage affecting the medium- and long-term prognosis of AMI patients.^3^

Previous studies have indicated that the prognosis of AMI patients after PCI depends not only on the efficacy of revascularization, but also on the quality of postoperative nursing quality, the level of continuous management and patient adherence.^4^,^5^ Conventional nursing models often lack standardization, early risk assessment and continuous intervention, making it difficult to meet the multidimensional, dynamically changing nursing needs of post-PCI patients.^6^ Furthermore, this group of patients is often prone to psychological stress responses such as anxiety and depression, which can further affect rehabilitation compliance and disease outcomes.^7^ Thus, there is a need to establish a more systematic, standardized and safety-oriented nursing management model.

The concept of Lean management, an approach that aims to improve the efficiency, quality and safety of healthcare services, has become increasingly popular in the nursing field.^8^ As a part of this approach, the 6S management model, a workplace organization methodology, adds a “Safety” element to the traditional 5S framework (Sort, Set in Order, Shine, Standardize, Sustain).^9^,^10^ This management model emphasizes reducing risk and improving efficiency through process standardization and continuous improvement.^9^,^10^ Studies have confirmed that 6S management can effectively improve nursing quality, reduce the incidence of adverse events and have a favorable impact on operating room nursing.^11^

Nevertheless, the efficiency of 6S management in the postoperative nursing of AMI patients undergoing PCI remains unclear. Previous research has shown that continuous nursing and systematic management improve functional recovery and quality of life in post-PCI patients, positively affect cardiopulmonary function and reduce the incidence of major adverse cardiovascular events after PCI.^12^–^14^ This study aimed to evaluate the impact of the 6S approach on clinical outcomes and nursing quality of AMI patients after PCI and to provide a new evidence-based basis for post-PCI nursing management.

## METHODOLOGY

Clinical records of patients with AMI who underwent PCI in the Second Hospital of Hebei Medical University from January 2024 to October 2025 were retrospectively analyzed. Patients who received the nursing scheme based on the 6S management mode were matched with the cohort who received the traditional nursing scheme at a ratio of 1:1. The matching conditions of the two groups were gender, age, body mass index (BMI), educational level, complications, smoking, classification of AMI, diseased vessels, presence of multivesicular disease, stent type and infarct size, etc.

### Ethical approval:

The study was approved by the Research Ethics Committee of the Second Hospital of Hebei Medical University (#2026-R035; dated January 23, 2026). Due to the retrospective nature of the study, the requirement for informed consent was waived.

### Inclusion criteria:

- Age ≥ 18 years old.
- Meets the International Society of Cardiology diagnostic criteria of AMI.
- AMI as the first diagnosis.
- PCI was performed through the radial artery approach.
- The clinical data are complete.

### Exclusion criteria:

- Cardiogenic shock.
- AMI was Killip III-IV.
- The existence of mental and other cognitive impairment.
- Severe renal insufficiency, liver insufficiency, severe chronic obstructive pulmonary disease, pulmonary tuberculosis or respiratory failure, hematopoietic system diseases, cancer and other serious primary diseases.

### Nursing Protocol:

Conventional nursing care for AMI after PCI included the following specific interventions:

- Given the sudden onset, rapid progression and the sense of impending death and fear caused by AMI, nurses conducted meticulous communication with the patients’ family members before surgery to alleviate fear and anxiety, thereby enabling them to actively cooperate with the treatment.
- On the 1st–2nd day after surgery, when patients were conscious or in stable condition, nurses were instructed to strengthen communication with them. Nurses used plain language to explain the methods, principles and efficacy of AMI treatment and cited successful cases to help patients build confidence in overcoming the disease, while relieving their tension, anxiety and depression.
- On the 3rd–8th day after surgery, nurses formulated individualized rehabilitation training plans for patients, covering the entire process from bed rest to ambulation to improve their postoperative quality of life and activities of daily living and further enhance their self-confidence.
- Patients were instructed to adopt a diet low in salt, fat, cholesterol and calories, but high in fiber and vitamins. A pattern of small, frequent meals was recommended to avoid postoperative digestive disorders.
- Before discharge, health education was provided to patients, focusing on the harms of unhealthy habits such as smoking and alcohol abuse. Patients were encouraged to develop healthy lifestyles, maintain a balance between work and rest and prevent postoperative cardiac adverse events.
- Family members were educated on AMI-related knowledge, including potential adverse events after PCI and their common clinical manifestations. This training aimed to ensure that family members could promptly identify such symptoms and seek medical attention for patients outside the hospital, avoiding delayed treatment.
- Family members were also advised to remind patients to take medications on time and return to the hospital regularly for follow-up examinations.

To ensure standardized execution, all participating nursing staff underwent a modular training program, consisting of four hours of intensive theoretical instruction followed by two weeks of supervised clinical bedside mentoring and proficiency assessment. The 6S management mode was implemented on the basis of the conventional nursing care and included:

### Sort

Core nursing tasks for patients after PCI were systematically sorted and classified to eliminate redundancy and optimize workflow. Nursing activities were categorized into emergency tasks (e.g., vital sign monitoring, puncture-site observation and arrhythmia surveillance), routine tasks (e.g., medication administration and basic life care) and long-term tasks (e.g., health education and rehabilitation guidance). Redundant records were removed, nursing documents were integrated and key clinical indicators, including myocardial injury markers, left ventricular ejection fraction (LVEF) and left ventricular end-diastolic dimension (LVEDD), were recorded in a unified format for improved documentation efficiency and accuracy.

### Set in order:

A graded nursing process table for AMI patients after PCI was developed to ensure orderly and standardized care delivery. Patients were stratified into low-, medium- and high-risk groups according to their clinical condition. For each risk level, the frequency of vital sign monitoring (e.g., every two hours for low-risk patients and every 30 minutes for high-risk patients), timing of ambulation and medication verification procedures were clearly defined, enabling precise and efficient nursing interventions.

### Shine

Ward cleanliness was ensured through daily collaboration between responsible nurses and cleaning staff. Medical contaminants were thoroughly removed and medical waste was strictly classified and disposed of in accordance with regulations. Ultraviolet disinfection was routinely performed, ward temperature was maintained at 22–25 °C and windows were regularly opened for ventilation to provide a clean, safe and comfortable treatment environment.

### Standardize

Standardized hygiene and basic care procedures were implemented based on patients’ postoperative recovery status. Low-risk patients received assistance with oral care and warm water body cleansing within 24 hours after surgery. Once vital signs of medium- and high-risk patients stabilized, family members were integrated into the “Standardize” protocol and instructed to assist with basic hygiene care, such as oral and skin maintenance, under nursing supervision to ensure continuity of care. These standardized practices ensured cleanliness of the skin, oral cavity and perineal area and helped prevent complications such as pressure injuries and oral infections.

### Sustain / Discipline:

Emphasis was placed on cultivating professional discipline and literacy among nursing staff. Training focused on standardizing clinical behaviors, enhancing communication skills, improving service etiquette and strengthening humanistic care awareness, particularly among undergraduate nurses. Nurses maintained continuous, bidirectional communication with patients and their families from admission to discharge to gain understanding, support, and trust. This ongoing collaborative process included comprehensive psychological assessments and timely emotional support to alleviate anxiety and improve treatment adherence.

### Safety (Security):

The principle of medical safety first was consistently upheld throughout nursing practice. Nurses closely monitored patients’ body temperature, blood pressure, heart rate, respiratory status, level of consciousness and pupillary responses. Special attention was paid to lung auscultation findings, including sputum sounds and moist rales. Clinical assessments were integrated with body temperature trends, sputum or blood culture results and chest X-ray findings. Abnormal changes were promptly reported to the attending physician to enable early intervention and reduce the risk of pulmonary and other postoperative complications.

### Collected indicators:

The following information were collected from clinical records:

- Demographic data (gender, education level) and clinical characteristics (BMI, comorbidity, smoking status, AMI classification, diseased vessels, number of diseased vessels, stent type, infarct size) were collected as baseline data.
- Rehabilitation-related indicators, specifically the length of hospital stay and the six-minute walk test (6MWT), were predefined as the primary endpoints of this study.
- Secondary and exploratory endpoints included other rehabilitation metrics (eating time and ambulation time), cardiac function indexes (LVEF and LVEDD), activities of daily living (assessed by the Barthel Index [BI]), anxiety status (assessed by the Self-Rating Anxiety Scale [SAS]), and the incidence of adverse cardiac events (angina pectoris, non-fatal myocardial infarction, severe arrhythmia, and congestive heart failure).

### Statistical analysis:

Statistical analyses were performed using the Windows version of SPSS/PC (Version 26.0; IBM Corp., Armonk, NY, USA). Categorical data were expressed as n (%). Intergroup differences were analyzed using the chi-square test or Fisher’s exact test, applied to variables including gender, comorbidities, educational level, smoking status, type of AMI, culprit vessel and stent type. The normality of continuous variables was assessed using visual methods (histograms and Q-Q plots) and analytical methods (Kolmogorov-Smirnov/Shapiro-Wilk tests). Normally distributed continuous data were presented as mean ± standard deviation (SD). Intergroup comparisons were conducted using independent-samples t-tests and intragroup comparisons between pre- and post-intervention were performed using paired t-tests; these methods were used for variables such as age, BMI, infarct size and 6MWT distance. Non-normally distributed continuous data were expressed as median and interquartile range (IQR). Intergroup comparisons were analyzed using the Mann-Whitney U test and intragroup comparisons between pre- and post-intervention were carried out using the Wilcoxon signed-rank test; these methods were adopted for variables including time to oral intake, time to postoperative ambulation and LVEF. Statistical significance was set at P < 0.05.

## RESULTS

**Fig.1:**
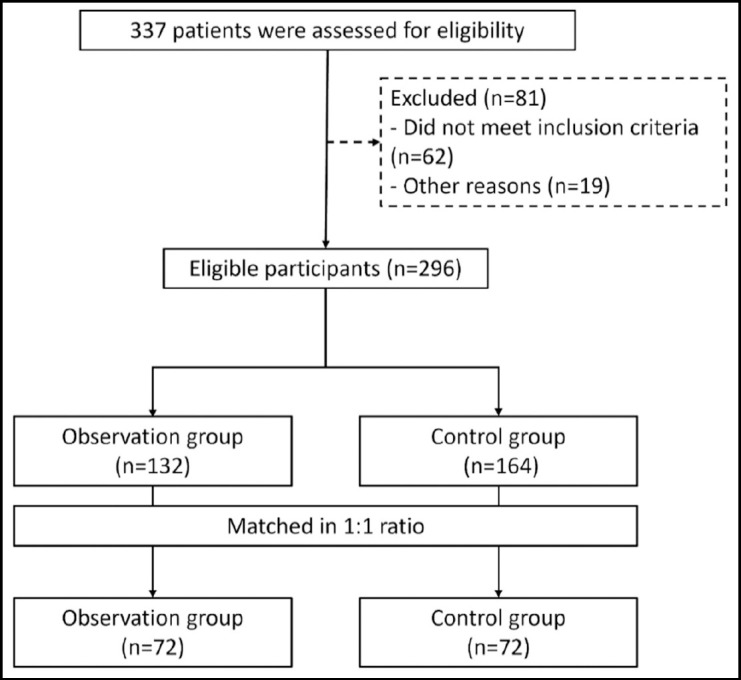
Study flow chart.

In this retrospective analysis, 377 AMI patients who underwent PCI were initially screened. A total of 81 patients were excluded: 37 did not meet the inclusion criteria and 23 met the exclusion criteria. Subsequently, 296 patients were enrolled in the study. Of them, 132 patients received nursing care based on the 6S management model and 164 received conventional nursing care. Propensity score matching (PSM) was performed to balance variables including gender, age, BMI, educational level, comorbidities, smoking status, AMI classification, culprit vessel, multivessel disease status, stent type and infarct size between the two groups. Patients were matched 1:1, with 72 cases in each group (Fig.1). The baseline characteristics of the patients are presented in Table-I; no statistically significant differences were observed between the two groups (P>0.05).

**Table-I:** Comparison of basic characteristics between the two groups.

Characteristics	Observation group (n=72)	Control group (n=72)	χ²/t	P
Male (yes),n(%)	55 (76.4)	59 (81.9)	0.674	0.412
Age (years)	57.6±8.3	59.2±9.3	-1.067	0.288
BMI (kg/m²)	24.0±2.6	23.4±2.8	1.291	0.199
Educational level,n(%)			2.887	0.089
Junior high school and below	24 (33.3)	34 (47.2)		
High school and above	48 (66.7)	38 (52.8)		
Hypertension (yes), n(%)	34 (47.2)	36 (50.0)	0.111	0.739
Diabetes (yes), n(%)	32 (44.4)	38 (52.8)	1.001	0.317
Hyperlipidemia (yes), n(%)	38 (52.8)	42 (58.3)	0.450	0.502
Smoker (yes), n(%)	39 (54.2)	35 (48.6)	0.445	0.505
AMI classification, n(%)			2.116	0.146
STEMI	54 (75.0)	61 (84.7)		
NSTEMI	18 (25.0)	11 (15.3)		
Culprit vessel, n(%)			0.838	0.658
Left anterior descending artery	37 (51.4)	38 (52.8)		
Left circumflex artery	15 (20.8)	11 (15.3)		
Right coronary artery	20 (27.8)	23 (31.9)		
Multivessel disease (yes), n(%)	37 (51.4)	28 (38.9)	2.271	0.132
Type of stent, n(%)			0.327	0.567
Sirolimus-eluting stent	52 (72.2)	55 (76.4)		
Everolimus-eluting stent	20 (27.8)	17 (23.6)		
Infarct size (%)	25.3±6.5	26.2±8.1	-0.759	0.449

The time to postoperative oral intake, time to ambulation, length of hospital stay and resolution of ipsilateral limb swelling in the observation group were significantly shorter than those in the control group, whereas the postoperative 6MWT distance was significantly longer (P<0.05). These findings indicated that the observation group had more favorable rehabilitation-related indicators than the control group.Table-II.

**Table-II:** Comparison of rehabilitation effects between the two groups.

Variables	Observation group (n=72)	Control group (n=72)	Z/t	P
Eating time (hours), median(IQR)	4 (3, 4.5)	4 (3, 5)	-	0.026
Ambulation time (hours), median(IQR)	5 (4, 5)	5 (4, 6)	-	0.033
Length of stay (days), median(IQR)	7 (6, 8)	9 (8, 10)	-	<0.001
Disappearance time of postoperative limb swelling (days), median(IQR)	3.5 (3, 5)	5 (4, 6)	-	<0.001
6MWT (m),mean±SD	367±48	329±55	4.737	<0.001

***Note:*** -, Mann-Whitney U test; 6MWT, 6-minute walk test; SD, standard deviation; IQR, interquartile range.

There were no statistically significant differences in baseline LVEF, LVEDD, BI and SAS scores between the two groups (P>0.05). Table-III. After the intervention, LVEF and BI levels in both groups were significantly higher than baseline, whereas LVEDD and SAS were significantly lower (P<0.05). Moreover, the observation group exhibited significantly higher LVEF and BI and significantly lower LVEDD and SAS, than the control group (P<0.05). These results demonstrated that both groups showed significant improvements in cardiac function, activities of daily living and anxiety levels compared with baseline and that the degree of improvement in the observation group was greater than that in the control group.

**Table-III:** Comparison of cardiac function, activities of daily living and anxiety between the two groups.

Variables	Observation group (n=72)	Control group (n=72)	Z	P
***Baseline***				
LEVF (%)	35 (34, 37.5)	34 (33, 37.5)	-	0.248
LVEDD (mm)	58 (55, 64.5)	58 (56, 62)	-	0.552
BI (score)	65 (58, 72.5)	67 (59.5, 74)	-	0.301
SAS (score)	54.5 (52, 62)	58 (52.5, 62)	-	0.257
***After intervention***				
LEVF (%)	45 (43, 49)*	42 (40, 46)*	-	<0.001
LVEDD (mm)	53 (50, 58)*	55 (52, 59)*	-	0.046
BI (score)	87 (85, 90)*	85 (78, 91.5)*	-	0.030
SAS (score)	41 (37, 45.5)*	46 (41, 51)*	-	<0.001

***Note:*** Compared with baseline level,

* P<0.05. LVEF, left ventricular ejection fraction; LVEDD, left ventricular end-diastolic diameter; BI, Barthel Index; SAS, Self-Rating Anxiety Scale. -, Mann-Whitney U test.

Compared with the control group, the observation group had a significantly lower incidence of cardiac adverse events after PCI, including angina pectoris and severe arrhythmia (P<0.05). Table-IV However, there was no statistically significant difference in the incidence of non-fatal myocardial infarction and ischemic heart failure between the two groups (P>0.05).

**Table-IV:** Comparison of the incidence of adverse cardiac events between the two groups.

Adverse event	Observation group (n=72)	Control group (n=72)	χ²	P
Angina pectoris, n(%)	6 (8.3)	15 (20.8)	4.516	0.034
Non fatal myocardial infarction, n(%)	4 (5.6)	10 (13.9)	2.848	0.091
Severe arrhythmia, n(%)	1 (1.4)	8 (11.1)	5.807	0.016^#^
Ischemic heart failure, n(%)	3 (4.2)	9 (12.5)	3.273	0.070

***Note:***

# Fisher’s Exact Test.

## DISCUSSION

This study investigated the impact of the nursing strategy based on the 6S management model on clinical outcomes in patients with AMI after PCI. The results showed that the 6S management-based nursing intervention was associated with more favorable postoperative rehabilitation-related indicators and clinical outcomes. These findings suggest that systematic nursing management may help optimize care processes and improve selected patient-centered outcomes in AMI patients after PCI.

Beyond statistical significance, the observed improvements in this study possess substantial clinical relevance for post-PCI recovery. The two day reduction in the median length of hospital stay (from 9 to 7 days) directly translates to enhanced hospital bed turnover rates and a tangible reduction in healthcare expenditures for patients.^15^,^16^ Furthermore, the 38-meter improvement in the 6-minute walk test (6MWT) distance exceeds the threshold for minimal clinically important difference (MCID) recognized in cardiovascular rehabilitation,^17^ suggesting that patients in the observation group achieved superior functional reserve and tolerance for daily activities upon discharge. The concurrent increase in Barthel Index scores implies that these patients can resume basic daily functioning earlier, effectively reducing the physical and emotional burden on family caregivers.^5^,^18^ From a psychological perspective, the reduction in Self-rating Anxiety Scale (SAS) scores signifies improved perioperative psychological resilience, which is a critical determinant of long-term adherence to secondary prevention medications and lifestyle modifications.^7^,^19^ Methodologically, the ‘Sort’ and ‘Set in Order’ components of the 6S model minimized redundant nursing tasks and optimized workflow,^10^,^16^ thereby allowing nursing staff to reallocate time toward high-value patient education and personalized rehabilitation guidance.

The success of the 6S management model in this study stems not only from professional nursing behaviors but also from the systematic synergy between nurses and family members.^20^ Rather than viewing family education as a one-time informational step, our protocol treated family participation as an ongoing collaborative process integrated into the 6S framework.^4^,^6^ In the ‘Standardize’ and ‘Safety’ phases, family members acted as active collaborators who assisted with basic hygiene and served as an additional layer of surveillance for early complication signs, such as puncture-site changes or recurrent chest pain.^20^ This high level of involvement likely increased family understanding and cooperation, which indirectly optimized patient outcomes. By fostering a supportive and organized environment, this collaborative approach significantly reduced patient anxiety (SAS scores) and facilitated the continuation of rehabilitation-related care from the hospital to the home setting, as evidenced by the improved 6MWT and BI scores.^13^,^21^

A critical consideration for any new nursing intervention is its scalability and resource requirements. The 6S model implemented in this study is essentially “resource neutral” and “efficiency-driven”.^8^,^22^ Rather than requiring additional staffing, the model focuses on eliminating “waste”—such as redundant documentation and time spent locating medical supplies—through the ‘Sort’ and ‘Set in Order’ phases.^9^,^16^ This optimization liberates nursing manpower, allowing for better focus on patient safety and education without increasing the overall workload. While ‘Safety’ and ‘Standardize’ are indispensable core elements for specialized cardiac centers, environmental tasks like ‘Shine’ can be simplified or delegated to auxiliary staff to suit resource-limited settings. Furthermore, the transition from intensive initial training to a three-tier quality control system (self-inspection, peer-review, and random audits) ensures that 6S behaviors eventually evolve into ingrained professional discipline (‘Sustain’), reducing long-term supervisory costs.^23^ These characteristics suggest that the 6S framework is highly adaptable to various clinical environments, including high-turnover wards where standardized workflows are paramount to preventing errors during rapid bed transitions.^15^,^22^

Previous studies have emphasized the importance of the postoperative nursing management model for ensuring a favorable prognosis of post-PCI patients. A randomized controlled trial by Yu et al.^14^ confirmed that structured management interventions can improve clinical safety outcomes after PCI, which is consistent with the results of this study and further supports the beneficial role of the 6S management concept in the comprehensive optimization of environment, processes and safety of post-PCI nursing care.

Previous studies have shown that fast-track nursing can shorten waiting time for emergency PCI and improve short-term outcomes in AMI patients,^15^ while holistic and refined nursing can enhance nursing satisfaction and reduce the incidence of complications.^24^,^25^ The 6S management-based nursing strategy in this study further emphasizes process standardization and continuous improvement. The observed clinical benefit may stem from systematic interventions in the nursing environment, staff behavior and risk control. Consistent with this study, Bu et al.^11^ reported that 6S management can significantly improve operating room nursing quality and reduce adverse events. Regarding safety outcomes, our results demonstrated a significant reduction in specific events such as angina pectoris and severe arrhythmia. However, no statistically significant differences were observed for harder clinical endpoints, including non-fatal myocardial infarction and heart failure. In nursing research, it is essential to distinguish between symptom-related outcomes and hard clinical events. The 6S management model likely improves the former through standardized monitoring, optimized workflow, and enhanced safety protocols, whereas demonstrating a significant impact on hard endpoints often requires larger sample sizes and more extended follow-up periods than provided in this retrospective study. Therefore, while the 6S model enhances perioperative safety and patient experience, its definitive role in preventing major hard cardiovascular events warrants further validation in larger prospective cohorts.

From a rehabilitation perspective, timely exercise rehabilitation and functional assessment have been shown to play important roles in the recovery of AMI patients after PCI, improving exercise tolerance and cardiopulmonary function.^18^,^26^,^27^ Systematic reviews and meta-analyses have further indicated that cardiac rehabilitation interventions help improve the quality of life of post-PCI patients and reduce the risk of readmission.^28^,^29^

Psychological and behavioral factors are also key determinants affecting the prognosis of AMI patients after PCI. Research has shown that negative emotions such as anxiety and depression can reduce treatment adherence and affect rehabilitation efficacy.^30^ A study by Mohandas et al.^19^ identified insufficient adherence to secondary prevention as an important predictor of myocardial infarction recurrence.

Earlier studies have shown that the concepts of 6S and Lean management can effectively improve the efficiency of medical processes and reduce the incidence of errors.^16^,^21^,^22^,^31^ Fang et al.^23^ confirmed that 6S nursing management can significantly improve work efficiency and quality control levels of central sterile supply departments.

### Limitations

First, the retrospective design makes the study inherently susceptible to recording bias and missing information. Although propensity score matching was utilized to balance major baseline characteristics, residual confounding remains possible; key factors such as symptom-to-reperfusion time, the consistency of perioperative pharmacotherapy, and baseline psychological status were not fully controlled, potentially influencing outcomes independently. Second, the relatively modest sample size and the broad range of outcomes evaluated without formal adjustment for multiple testing may have increased the risks of both Type-I and Type-II errors. The observed differences in the primary rehabilitation-related indicators, including length of hospital stay and 6MWT, were relatively clear in this sample; however, the study may have been underpowered to detect differences in low-frequency adverse cardiac events, such as non-fatal myocardial infarction and heart failure. Therefore, the adverse event findings should be regarded as exploratory and interpreted with caution. Third, as a single-center study conducted in a large tertiary hospital with a mature nursing team and a robust culture of standardization, the success of the 6S model may be tied to specific organizational capacities. The high professional discipline of the staff—particularly undergraduate nurses—was a critical determinant for the ‘Sustain’ phase, suggesting that the model’s impact and sustainability could vary in settings with different staffing structures or lower managerial maturity. Finally, the short follow-up duration limits our ability to interpret the long-term clinical impact and sustainability of the intervention once patients return to their communities. Consequently, the definitive long-term clinical superiority of this model remains to be established through future multi-center, prospective, randomized controlled trials across diverse healthcare systems.

## CONCLUSION

Implementing the nursing strategy based on the 6S management model for AMI patients after PCI helps standardize nursing processes, strengthen safety management, and improve nursing quality. It helps optimize patients’ postoperative rehabilitation outcomes and enhance the effectiveness of nursing management. This nursing model is guided by systematic, standardized management principles and offers a feasible and effective approach to the comprehensive care of post-PCI patients. Future studies are still needed to further verify its impact on patients’ long-term prognosis through multi-center research across diverse organizational cultures and healthcare systems, so as to provide a more comprehensive evidence-based basis for clinical nursing practice.
